# Lack of Type I Interferon Signaling Ameliorates Respiratory Syncytial Virus-Induced Lung Inflammation and Restores Antioxidant Defenses

**DOI:** 10.3390/antiox11010067

**Published:** 2021-12-28

**Authors:** Maria Ansar, Yue Qu, Teodora Ivanciuc, Roberto P. Garofalo, Antonella Casola

**Affiliations:** 1 Department of Pediatrics, The University of Texas Medical Branch, Galveston, TX 77555, USA; maansar@utmb.edu (M.A.); yuqu@utmb.edu (Y.Q.); teivanci@utmb.edu (T.I.); rpgarofa@utmb.edu (R.P.G.); 2 Department of Microbiology and Immunology, The University of Texas Medical Branch, Galveston, TX 77555, USA; 3 Institute for Human Infections and Immunity, The University of Texas Medical Branch, Galveston, TX 77555, USA

**Keywords:** respiratory syncytial virus, inflammation, antioxidant enzymes, NRF2, PML

## Abstract

Respiratory syncytial virus (RSV) infection in mouse and human lung is associated with pathogenic inflammation and oxidative injury. RSV impairs antioxidant responses by increasing the degradation of transcription factor NF-E2-related factor 2 (NRF2), which controls the expression of several antioxidant enzymes (AOEs). In addition to its protective effects, type I IFNs have been increasingly recognized as important mediators of host pathogenic responses during acute respiratory viral infections. We used a mouse model of RSV infection to investigate the effect of lack of type I interferon (IFN) receptor on viral-mediated clinical disease, airway inflammation, NRF2 expression, and antioxidant defenses. In the absence of type I IFN signaling, RSV-infected mice showed significantly less body weight loss and airway obstruction, as well as a significant reduction in cytokine and chemokine secretion and airway inflammation. Lack of type I IFN receptor was associated with greatly reduced virus-induced promyelocytic leukemia lung protein expression, which we showed to be necessary for virus-induced NRF2 degradation in a cell model of infection, resulting in restoration of NRF2 levels, AOE expression, and airway antioxidant capacity. Our data support the concept that modulation of type I IFN production and/or signaling could represent an important therapeutic strategy to ameliorate severity of RSV-induced lung disease.

## 1. Introduction

Type I interferons (IFNs) are a major component of innate host defense against viral infections. At the early phase of viral infection, rapid production of type I IFNs by various cell types leads to expression of IFN-stimulated genes that limit viral replication and spread [1]. As such, loss of IFN type I signaling can usually be associated with uncontrolled viral replication in human natural infections and in experimental animal models [2]. However, in addition to its protective effects, type I IFNs have been increasingly recognized as important mediators of host pathogenic responses during acute respiratory viral infections, such as those caused by influenza viruses, in the context of both naturally acquired and experimental human infections [3,4,5]. Although some of the pathogenic effects triggered by type I IFNs during viral infections have been described, including enhanced cytokine production and inflammation, epithelial cell damage by apoptosis, or T-cell-mediated cytotoxicity, other IFN-dependent pathways which could be critical determinants of disease severity have yet to be characterized (reviewed in [6,7]). Respiratory syncytial virus (RSV) is the most important cause of viral acute respiratory tract infections and hospitalizations in children, for which no vaccine or specific treatment is available. RSV causes airway mucosa inflammation and cellular oxidative damage by triggering production of reactive oxygen species and by concurrently inhibiting expression of antioxidant enzymes (AOEs), via degradation of the transcription factor NF-E2-related factor 2 (NRF2) [8,9,10]. In a recent in vitro investigation, we found that RSV-induced NRF2 degradation occurs in a RING finger protein 4 (RNF4)–promyelocytic leukemia (PML)-dependent manner. Inhibition of PML expression resulted in a significant reduction of RSV-associated NRF2 degradation and increased antioxidant enzyme expression, identifying PML as a key regulator of antioxidant defenses in the course of RSV infection [11]. In this study, we investigated the role of type I IFN signaling in RSV-induced body weight loss, airway obstruction, lung inflammation, and antioxidant pathways using an in vivo model of infection. Our results show that lack of type I IFN signaling significantly ameliorates the abovementioned parameters of clinical disease and reduces lung inflammation. They also show for the first time that lack of type I IFN signaling is associated with decreased viral-induced PML expression and enhanced levels of NRF2 in the airways, resulting in restoration of AOE expressions and antioxidant capacity in the lungs.

## 2. Materials and Methods

### 2.1. Ethics Statement

All procedures involving mice in this study were performed in accordance with the recommendations in the Guide for the Care and Use of Laboratory Animals of the National Institutes of Health. Pathogen-free breeding colonies of WT mice were maintained at the Animal Resource Center facility, University of Texas Medical Branch at Galveston. A mixture of ketamine (90–150 mg/kg) and xylazine (7.5–16 mg/kg) was administered by intraperitoneal (IP) injection for anesthesia and euthanasia. The protocol was approved by the Institutional Animal Care and Use Committee of the University of Texas Medical Branch at Galveston (Protocol: 9001002).

### 2.2. Animal Studies and RSV Infection Protocol

The RSV Long strain was grown in HEp-2 cells and purified on discontinuous sucrose gradients, as previously described [12]. Viral titers were determined by plaque-forming units (PFU/mL) using a methylcellulose plaque assay, as previously described [13]. Male and female mice deficient for alpha/beta interferon receptor (IFNAR KO), on the 129/Sv background, lacking subunit 1 of the receptor [2], were generously provided by Herbert Virgin at the Washington University School of Medicine, St. Louis, MO. The control mice of the parental strain (129S1/SvImJ) were purchased from Jackson Laboratory (Bar Harbor, ME, USA). Pathogen-free breeding colonies of IFNAR KO and WT mice were maintained at the University of Texas Medical Branch at Galveston and experiments were performed using 12 to 16 week old mice of both genders (male-to-female ratio 1:1). Mice were infected intranasally with 1 × 10^7^ PFU of RSV in 50 µL of phosphate-buffered saline (PBS). As mock treatment, mice were treated as above and inoculated with an equivalent volume of PBS. Each experimental condition included 4–6 mice/group. Clinical disease, evaluated by body weight loss, airway obstruction, lung inflammation, and viral replication, was used as the outcome, as previously described [14].

### 2.3. Airway Function Parameters

Airway obstruction was determined utilizing whole-body barometric plethysmography (BuxcoTM, DSI, New Brighton, MN, USA) [15]. Baseline enhanced pause (Penh), a dimensionless value measured as a ratio of peak expiratory flow to inspiratory flow over the time of expiration, was measured on days 1, 3, and 5 post infection (p.i.), as previously described [16].

### 2.4. Differential Cell Count, Cytokine, and Chemokine Analysis

Mice were euthanized, and bronchoalveolar lavage (BAL) was collected at the indicated timepoints. An aliquot was used for cytospin analysis, and the remainder was stored at −80 °C for cytokine and chemokine analysis. Total cell counts were determined via a hemacytometer, and viability was determined through trypan blue. Cytospin slides were stained with HEMA3 (Fisher Scientific, Hampton, NH, USA), and differential cell counts were determined via light microscopy. Cell counts were determined at 6 h, as well as on days 1, 2, and 5 p.i. Levels of cytokines and chemokines were measured in BAL fluid (BALF) at the same timepoints using a Bio-Plex Pro Mouse Group I 23-plex panel kit (Bio-Rad Laboratories, Hercules, CA, USA). Levels of TGF-β1, TGF-β2, and TGF-β3 in BAL fluid were determined with the Bio-Plex Pro TGF-β 3-Plex (Bio-Rad Laboratories, Hercules, CA, USA) and the level of IL-33 was determined with a specific ELISA kit (Invitrogen, Thermo Fisher Scientific, Waltham, MA, USA). IFN-α, IFN-β, and IFN-λ 2/3 were measured using commercial enzyme-linked immunosorbent assays (ELISAs), following the manufacturer’s protocol (PBL Biomedical Laboratories, Piscataway, NJ, USA). The sensitivity ranges of the assays were 12.5–400 pg/mL for IFN-α, 12.5–1000 pg/mL for IFN-β, and 31.2–2000 pg/mL for IFN-λ 2/3.

### 2.5. Lung Histopathology

On day 7, mice were euthanized with an intraperitoneal injection (IP) of ketamine and xylazine followed by exsanguination via the femoral vessels. Lung tissues were fixed in 10% buffered formalin followed by paraffin embedding. Multiple 4 µm longitudinal cross-sections were stained with hematoxylin and eosin (H&E). The slides were analyzed under light microscopy.

### 2.6. Western Blot

Total lung extracts were prepared using RIPA buffer. Equal amounts of protein (100 µg per sample) were boiled in 2× Laemmli buffer and resolved on SDS-PAGE gels. Proteins were transferred onto a polyvinylidene difluoride membrane (Amersham, Piscataway, NJ, USA), and nonspecific binding sites were blocked in Tris-buffered saline (TBS) containing 5% skim milk powder. Membranes were incubated with the primary antibody at the recommended dilution overnight at 4 °C, followed by incubation with horseradish peroxidase (HRP)-conjugated secondary antibody for 1 h at room temperature. Proteins were detected using an enhanced chemiluminescence system (RPN 2016; Amersham, GE Healthcare, United Kingdom). Membranes were stripped and re-probed with anti-β-actin antibody (Sigma-Aldrich, St. Louis, MO, USA) for loading control. Densitometric analysis of band intensities was performed using UVP VisionWorksLS Image Acquisition and Analysis Software 8.0 RC 1.2 (UVP, Upland, CA, USA). Primary antibodies used for Western blot assays were PML (cat#05-718; Millipore Sigma, Burlington, MA, USA) and NRF2 (cat#16396-1-AP; Proteintech, Rosemont, IL, USA). As the observed mouse NRF2 bands affected by RSV infection were of a lower molecular weight than expected for activated human NRF2, specificity was determined by neutralizing the Proteintech antibody with the recombinant NRF2 protein fragment (cat#16396-1-AP). Both the 75 and 52 kDa bands were specific, as they were no longer detectable by the neutralized antibody. 

### 2.7. Real-Time PCR (RT-PCR)

Total RNA was extracted from whole lung using the ToTALLY RNA kit from Ambion (Cat # AM1910, Austin, TX, USA). Synthesis of cDNA was performed with 1 µg of total RNA in a 20 µL reaction using the reagents in the Taqman Reverse Transcription Reagents Kit from ABI (Applied Biosystems #N8080234), according to the manufacturer’s instructions. Quantitative PCR amplification was done using 1 µL of cDNA in a total volume of 25 µL using the Faststart Universal SYBR green Master Mix (Roche Applied Science #04913850001). The final concentration of the primers was 200 nM. 18S RNA was used as a housekeeping gene for normalization. PCR assays were run in the ABI Prism 7500 Sequence Detection System. Primer sequences are available upon request.

### 2.8. Hydroxyl Radical Antioxidant Capacity Measurement

Hydroxyl radical antioxidant capacity (HORAC) was determined in BALF samples using an assay kit from Cell Biolabs (San Diego, CA, USA) and analyzed using the FLUOStar Optima (BMG Labtech, Cary, NC, USA).

### 2.9. Advanced Oxidation Protein Product (AOPP) Measurement

AOPPs are a group of oxidatively modified protein products generated by reactive oxygen species or formed during oxidative/chlorine stress [17]. AOPPs in BAL were determined using a spectrophotometric method based on the reaction between chloramine reaction initiator and AOPP in unknown AOPP-containing samples. Briefly, 200 uL of BAL diluted 1:4 in PBS and 200 uL of different concentrations of chloramine-T solutions in PBS as a standard were placed in a 96-well plate. Ten microliters of 1.16 M KI (potassium iodine in PBS) was added, followed by 20 µL of glacial acetic acid. The yellow color was read at 340 nm in a microplate reader. The AOPP content was determined through comparison with the chloramine standard curve. The concentration of the AOPP was expressed in μM of chloramine-T.

### 2.10. Statistical Analysis

All experiments were repeated a minimum of two times, using 4–6 animals/group and a different viral pool for each experiment. Data are presented as averages with standard deviations. Statistical analysis was performed using GraphPad Prism 8.2 (GraphPad Software, Inc., San Diego, CA, USA). Statistical tests included repeated-measures ANOVA, one-way ANOVA, and Student’s *t*-test.

## 3. Results

### 3.1. Lack of IFN I Receptor Is Associated with Improved RSV Disease in Mice

**Figure 1:**
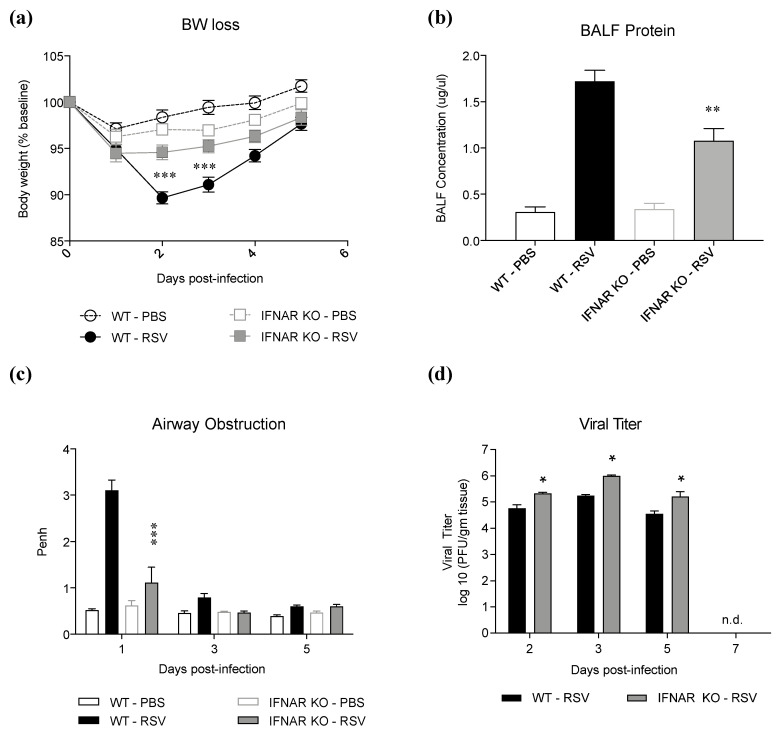
Effect of IFN I receptor knockout on disease parameters in RSV infection. IFNAR KO and WT mice were infected with and assessed for (**a**) body weight loss, (**b**) BALF protein levels on day 2 p.i., (**c**) airway obstruction, and (**d**) viral titer on different days p.i. Data are shown as means ± SD. Significance was determined using repeated-measures ANOVA for (**a**) and (**c**), one way ANOVA for (**b**), and Student’s *t*-test for (**d**); * *p* < 0.05, ** *p* < 0.01, and *** *p* < 0.001 compared to WT RSV. Data are representative of one of two independent experiments, with 4–6 animals/group.

As type I IFN production is associated with both protective and pathogenic responses in models of viral infections, we investigated whether lack of type I IFN signaling would confer protection against RSV infection. IFNAR KO and WT mice were infected with RSV and evaluated for body weight loss, airway obstruction, lung damage, and viral replication at different timepoints of infection. Lack of IFN type I receptor resulted in a significant amelioration of RSV-induced body weight loss (Figure 1a), and it was associated with a significant reduction in BALF total protein levels, a measure of epithelial barrier damage (Figure 1b). IFNAR KO mice also displayed a significant reduction in RSV-induced airway obstruction (i.e., basal Penh values), measured by whole-body plethysmography (Figure 1c), on day 1 p.i., which represents the peak day of obstruction in the course of infection. IFNAR KO infected mice showed a consistent but limited increase in lung viral titer throughout the course of infection, without prolonged shedding (Figure 1d), in agreement with the known limited role of type I IFN in controlling RSV replication [18]. 

### 3.2. Lack of IFN I Receptor Inhibits RSV-Induced Lung Inflammation

**Figure 2:**
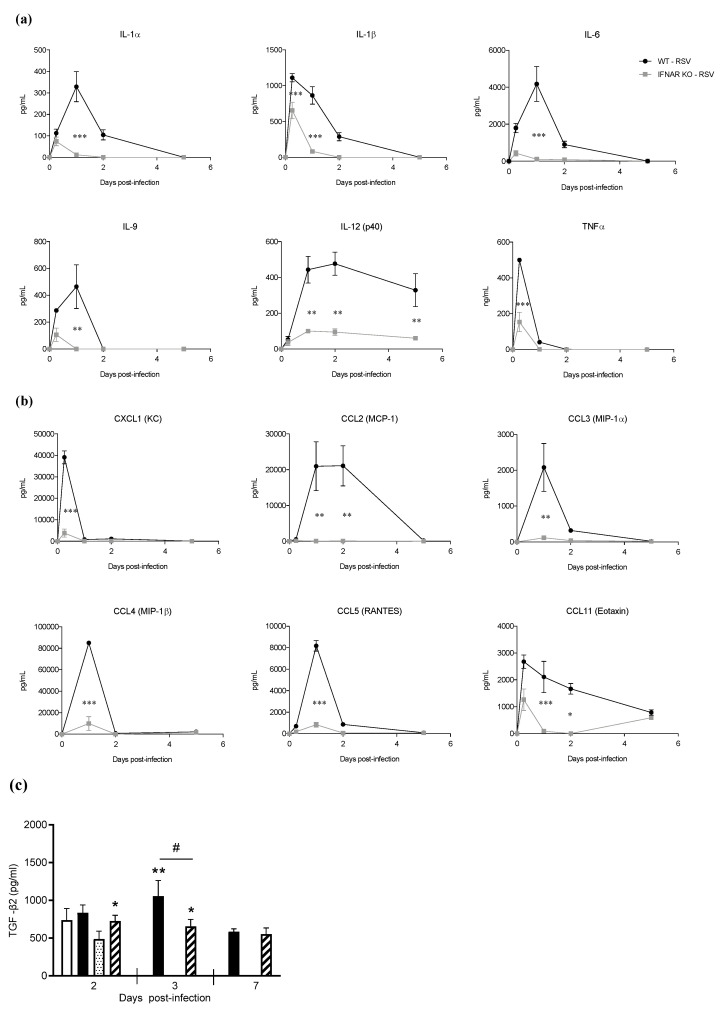

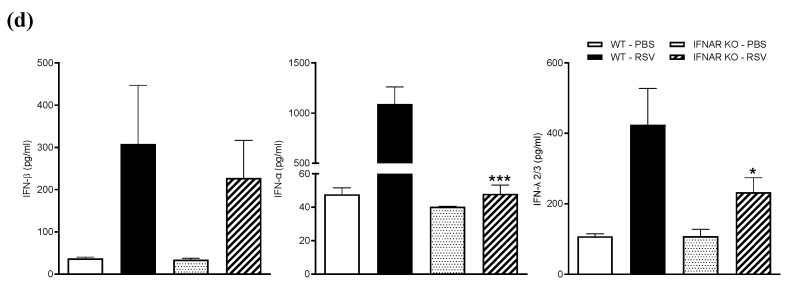
Proinflammatory mediator response in IFN I receptor knockout. IFNAR KO and WT mice were infected with RSV and sacrificed at different timepoints of infection to collect BAL for measurements of cytokines (**a**), chemokines (**b**), TGF-β (**c**), and type I and III IFN (**d**). Data are shown as means ± SD. Significance was determined using repeated-measures ANOVA for (**a**), (**b**), and (**c**), and Student’s *t*-test for (**d**); * *p* < 0.05, ** *p* < 0.01, and *** *p* < 0.001 compared to WT RSV with the exception of 2c, where it indicates a difference between RSV and PBS mice. # *p* < 0.05 compared to WT RSV. Data are representative of one of two independent experiments, with 4–6 animals/group.

As inflammation is an important component of RSV-induced lung disease pathogenesis, we investigated BALF cytokine and chemokine levels and inflammatory cell recruitment in IFNAR KO and WT mice at different timepoints of infection. Lack of IFN I receptor resulted in a significant reduction in RSV-induced secretion of the cytokines IL-1α, IL-1β, IL-6, IL-9, IL-12 (p40), and TNF-α (Figure 2a). Levels of several proinflammatory chemokines, namely, CXCL1, CCL2, CCL3, CCL4, CCL5, and CCL11, were also significantly reduced in the KO mice compared to the WT (Figure 2b). Among the Th2-skewing cytokines, there was no significant induction of IL-4, IL-5, and IL-33 in response to RSV infection in both IFNAR KO and WT mice at all timepoints tested (data not shown), while there was a limited increase in TGF-β2 secretion at the earlier timepoints of infection, which was lower in the IFNAR KO mice compared to the WT (Figure 2c). When we measured type I and III IFN BALF levels, there was no significant difference in IFN-β secretion, while IFN-α and -λ were both significantly reduced in the IFNAR KO mice compared to the WT (Figure 2d).

**Figure 3:**
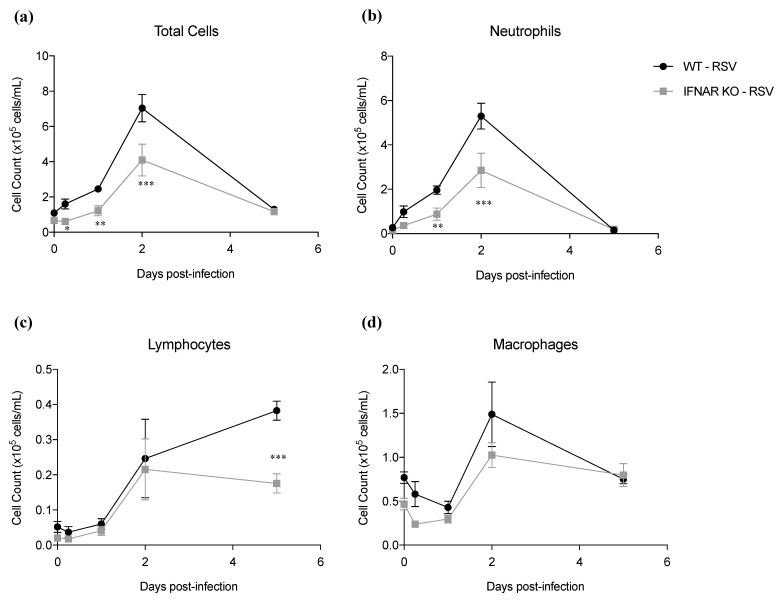

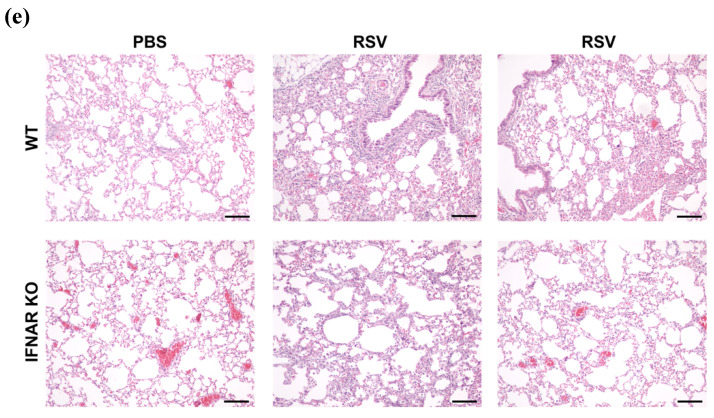
Effect of IFN I receptor knockout on BALF cell recruitment and lung histopathology. IFNAR KO and WT mice were infected with RSV and sacrificed at different timepoints of infection to collect BAL for total and differential cell count analysis; numbers of (**a**) total cells, (**b**) neutrophils, (**c**) lymphocytes, and (**d**) macrophages/monocytes were determined. Data are shown as means ± SD. Significance was determined using repeated-measures ANOVA; * *p* < 0.05, ** *p* < 0.01, and *** *p* < 0.001 compared to WT RSV. Data are representative of one of two independent experiments, with 4–6 animals/group for BALF studies. (**e**) For histopathology, three mice/group were sacrificed on day 7, lungs were embedded in paraffin for sectioning, and slides were stained with H&E. Bars = 100 µm.

We subsequently determined inflammatory cell recruitment in BAL from IFNAR KO and WT infected mice. Lack of IFN I receptor resulted in a significant reduction in BAL total cell number (Figure 3a). For differential cell count, both neutrophils (Figure 3b) and lymphocytes (Figure 3c) were lower in IFNAR KO *RSV*-infected mice, compared to WT, while levels of macrophages did not change significantly (Figure 3d), in agreement with a significant reduction in chemokine secretion observed in IFNAR KO mice. Lung histopathology performed on day 7 after RSV inoculation showed that IFNAR KO mice had an overall reduction in the percentage of parenchyma involved and peribronchial cuffing compared to WT mice (Figure 3e).

### 3.3. Lack of IFN I Receptor Is Associated with Rescue of NRF2 Degradation and Restoration of Antioxidant Defenses in the Lungs

**Figure 4:**
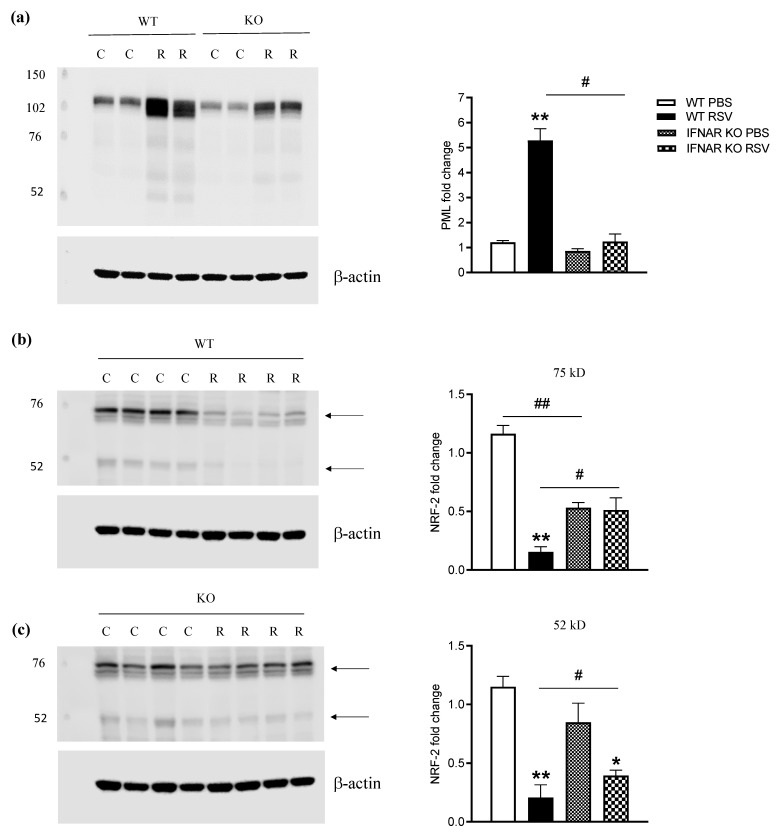
Effect of IFN I receptor knockout on PML and NRF2 AOE expression. Total lung lysates from mice that were either sham PBS-inoculated (C) or infected with RSV (R) for 48 h were subjected to Western blot analysis with anti-PML (**a**) or -NRF2 (**b**,**c**) antibodies. For loading controls, membranes were stripped and reprobed with anti-β-actin antibody. Densitometric analysis of band intensity, normalized first to β-actin and then to WT PBS, is shown in the right graph panels. Arrows indicate lower- and higher-molecular-weight forms of NRF2. Data are expressed as means ± SD. Significance was determined using Student’s *t*-test; ** *p* < 0.01 and * *p* < 0.05 relative to PBS-inoculated mice; ^#^ *p* < 0.05 relative to WT RSV-infected mice; ^##^ *p* < 0.05 relative to WT PBS mice. Data are representative of one of two independent experiments, with 4–6 animals/group.

PML protein is a member of the TRIM family and a major component of PML nuclear bodies (NBs). PML-NBs are involved in a wide variety of cellular processes, through facilitation of post-translational modifications of partner proteins, resulting in partner sequestration, activation, or degradation [19]. In the past few years, we have shown that RSV causes airway mucosa inflammation and cellular oxidative damage by triggering production of reactive oxygen species and by simultaneously inhibiting the expression of antioxidant enzymes, via degradation of the transcription factor NRF2, which occurs in an RNF4-PML-dependent manner [11]. PML is primarily upregulated over the course of infections by type I and type II interferons [20,21]. Therefore, to determine the impact of the lack of type I IFN signaling on the PML/NRF2/AOE axis, IFNAR KO and WT mice were infected with RSV for 48 h and sacrificed to prepare total lung extracts or total RNA. IFNAR KO mice showed a significant reduction in RSV-induced lung PML expression compared to WT mice (Figure 4a). Although NRF2 protein levels in PBS-treated/uninfected IFNAR KO mice were lower than in WT animals, we did not observe RSV-induced NRF2 degradation in IFNAR KO mice when compared to WT mice (Figure 4b,c).

**Figure 5:**
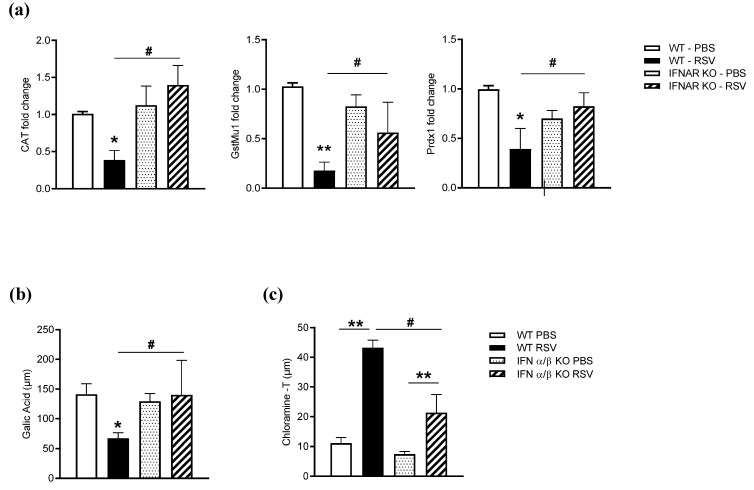
Effect of IFN I receptor knockout on AOE expression, antioxidant capacity, and markers of oxidative stress. Mice either sham PBS-inoculated or infected with RSV for 48 h were sacrificed to extract total lung RNA. Catalase, GST, and Prdx1 gene expression was quantified by q-RT-PCR (**a**). Data are expressed as means ± SD and normalized to WT PBS. Statistics were determined using Student’s *t*-test; ** *p* < 0.01 and * *p* < 0.05 relative to PBS-inoculated mice; ^#^ *p* < 0.05 relative to WT RSV-infected mice. BALF was collected on day 2 p.i. to measure (**b**) hydroxyl radical antioxidant capacity (gallic acid assay) and (**c**) advanced oxidation protein products (chloramine-T assay). Data are expressed as means ± SD. Statistics were determined using Student’s *t*-test; ** *p* < 0.01 and * *p* < 0.05 relative to PBS-inoculated mice; ^#^ *p* < 0.05 relative to WT RSV-infected mice. Data are representative of one of two independent experiments, with 4–6 animals/group.

As result of a lack of NRF2 degradation, expression of AOEs was significantly preserved in RSV-infected IFNAR KO compared to WT mice (Figure 5a). Similarly, the airway antioxidant capacity was not significantly reduced in IFNAR KO mice compared to WT following RSV infection (Figure 5b). Moreover, advanced oxidation protein products (AOPPs), an established biomarker of oxidative stress in biological fluids, were significantly increased in BAL of RSV-infected mice as compared to uninfected ones, but levels were significantly lower in IFNAR KO mice compared to WT (Figure 5c).

## 4. Discussion

Type I IFNs function in both autocrine and paracrine manners to induce the expression of various interferon-stimulated genes (ISGs) that confer antiviral activities to host cells, although many species of viruses, including RSV, have evolved mechanisms to evade IFN antiviral function. On the other hand, type I IFNs have been increasingly recognized as important mediators of pathogenic responses during acute respiratory viral infections. In the past several years, there has been published evidence suggesting that IFN type I responses are associated with disease severity in naturally acquired RSV infections. Few clinical studies have shown that both (airway) mucosal and systemic IFN I responses are associated with increased severity of lower respiratory tract infections in infants and young children [22,23,24]. In one study, IFN I gene expression/dysregulation was associated with acute neutrophilia, and it persisted as long as 1 month after resolution of the infection [23]. Although this clinical evidence supports the concept that IFN type I could be involved in the pathogenesis of RSV bronchiolitis, the downstream mechanisms that mediate disease are not fully known. A recent study using IFNAR KO mice provided evidence that, in RSV-infected mice, type I IFN receptor signaling amplifies early proinflammatory cytokine production in the lung and, therefore, could be implicated in disease pathogenesis [25]. Lack of type I IFN signaling was associated with reduced INF-α and -λ production and an increase in viral replication, which the authors indicated as the cause of worsened body weight loss during infection. In agreement with that study, our results show that RSV-infected mice genetically deficient in the IFNAR display significantly reduced proinflammatory cytokine and chemokine secretion in the airways, resulting in reduced BAL inflammatory cell recruitment, specifically neutrophils, mostly present in the first few days post infection, and lymphocytes, at later timepoints. We also observed milder pathological features of RSV-induced lung inflammation in IFNAR KO mice, although those studies were not performed in the abovementioned study. Like in Goritzka’s paper, we found that blocking type I IFN signaling affected production of IFN-α and INF-λ. Induction of IFN-β precedes IFN-α chronologically and does not require signaling through IFNAR; binding of IFN-β to IFNAR induces subsequent transcriptional responses leading to IFN-α expression. IFN-β signaling also amplifies IFN-λ production, although INF-λ, like IFN-β, is a primary response following viral infections [26].

Unlike Goritzka’s paper, we observed a significant improvement in several parameters of clinical disease, as shown by a decrease in body weight loss, airway obstruction, and BALF protein content, even in the presence of a moderate increase in viral replication. Differences in our results could be due to mouse background strain (129/Sv vs. C57BL6), virus strain (Long vs. A2 strain), purification of the virus (purified vs. stock), and inoculum (high vs. low). Our experimental mouse model of infection is associated with a more robust disease at earlier timepoints of infection, during activation of innate responses, compared to later times, when weight loss and lung pathology coincide with the infiltration of T cells [27]. Similar to RSV, there have been discrepancies regarding the role of type I IFN in restricting influenza infection in vivo and its role in pathogenesis, with some studies suggesting a protective role while others found no effect. Possible reasons for the discrepancies between these studies are infection with different virus doses and strains, as well as mouse backgrounds, making comparisons between studies difficult. Using the influenza virus strains A/PR/8/34 (H1N1) and X31 (H3N2), as well as IFNAR KO mice in the 129/SvEv background, Price et al. found no major effect on overall lethality, virus replication, the kinetics of the cellular immune response, or the ability to respond to subsequent virus challenge [28]. In a similar model of infection, with lower inoculum, lack of the type I IFN receptor was found to reduce morbidity and mortality, indicating a contribution of type I IFN to disease [29]. No other respiratory viruses have been as extensively investigated as influenza; however, infection with 10^5^ PFU of human metapneumovirus of IFNAR KO mice in the C57BL6 background did not result in enhanced disease during an extended period of observation [30].

Type III IFN (INF-λ) responses, unlike type I, are primarily restricted to mucosal surfaces and are thought to confer antiviral protection without driving damaging proinflammatory responses [31]. During infection with respiratory viruses, disease severity is linked to lung epithelial destruction, due to both cytopathic viral effects and immune-mediated damage. Recent studies have shown that IFN signaling interferes with lung repair during respiratory virus infections, such as influenza, in mice, by reducing epithelial proliferation and differentiation, which increases disease severity and susceptibility to bacterial superinfections, with IFN-λ driving these effects most potently [32]. The reduction of INF-λ production in IFNAR KO mice, compared to WT, in response to RSV infection could have contributed to the observed increase in viral replication; however, it could have also played a role in the reduction in BALF total protein levels, a measure of epithelial barrier damage, as well as to the amelioration of body weight loss. A recent study of children hospitalized with RSV bronchiolitis showed a positive correlation between transcript levels of IFN-λ and a clinical score index, although the study did not include assessment of protein levels or expression of IFN type I [33].

Although our understanding of the mechanisms that determine the severity of lower-respiratory-tract infections caused by RSV is still incomplete, several recent studies have indicated an important role of reactive oxygen species (ROS) produced by epithelial and inflammatory cells, as well as subsequent oxidative stress, in the pathogenesis of RSV infection, as inhibiting ROS production by administering antioxidants significantly decreases lung injury and improves clinical disease [9]. RSV infection induces oxidative stress, both in vitro and in vivo. AOE gene transcription is regulated through binding of NRF2 to the antioxidant responsive element (ARE) site located in its promoter [30]. While other viruses associated with ROS production have been shown to induce ARE-dependent responses by activating NRF2, including hepatitis B and C viruses, human cytomegalovirus, Kaposi’s sarcoma-associated herpes virus, and Marburg virus [34,35,36], we were the first to show that RSV infection is uniquely associated with a progressive reduction in NRF2 nuclear and cellular levels, leading to inhibition of ARE-dependent gene expression. PML nuclear bodies (NBs) are membrane-free subnuclear compartments whose formation is initiated and controlled by the PML protein, a member of the tripartite motif (TRIM) family, which is upregulated in viral infections by type I and type II interferons [20,21]. In recent studies, we found that RSV infection induces PML expression and PML-NB formation in airway epithelial cells in an IFN-dependent manner. In the absence of IFN-dependent signaling, there was no induction of PML protein in response to RSV infection, and there was a significant reduction in NRF2 degradation, resulting in enhanced antioxidant gene expression. Our results, using the IFNAR KO mice, showing that lung NRF2 levels and, in turn, AOE expression were preserved in the KO mice compared to WT mice, leading to lung antioxidant capacity levels that were comparable to those of uninfected mice, strongly support the hypothesis that IFN type I, via activation of PML, causes degradation of NRF2 and impaired expression of AOE, a novel and previously undescribed mechanism of IFN pathogenicity in RSV infection. 

## 5. Conclusions

Altogether, our study further supports the concept that modulation of type I IFN production/signaling could represent an important therapeutic strategy to ameliorate the severity of RSV-induced lung disease by limiting airway inflammation and counteracting virus-induced oxidative damage.

## Data Availability

Data is contained within the article.
